# Measurement properties of health-related quality of life instruments for people living with scoliosis in China: A systematic review

**DOI:** 10.1371/journal.pone.0325382

**Published:** 2025-06-26

**Authors:** Liu-qing Jing, Qi-xing Shen, Yi-lin Jin, Shao-jie Du, Ting-zhen Liu, Ni Chen, Guo-dong Wang, Wen Mo, Jie Ye, Jin-hai Xu

**Affiliations:** 1 Department of Orthopaedics, Longhua Hospital Affiliated to Shanghai University of Traditional Chinese Medicine, Shanghai, China; 2 Shanghai Spinal Diseases Institute of Traditional Chinese Medicine, Shanghai, China; Iran University of Medical Sciences, IRANISLAMIC REPUBLIC OF

## Abstract

**Purpose:**

Scoliosis is a three-dimensional structural deformity of the spine that leads to restricted spinal motion, weakness in the paraspinal muscles, reduced cardiopulmonary function, and chronic physical and psychological pain. It also impacts patients’ quality of life. According to statistics, as of 2022, the number of Chinese primary and secondary school students with scoliosis has exceeded 5 million, and continues to grow at an annual rate of approximately 300,000 new cases, while the incidence of scoliosis in adults is even higher. Therefore, it is crucial to evaluate health-related quality of life (HR-QoL) in this population. This systematic review examined the frequency of use of HR-QoL outcome measurement instruments among patients with scoliosis in China and synthesized evidence on their measurement properties.

**Methods:**

Stage 1: We searched databases including MEDLINE, EMBASE, Scopus, PubMed, and the Web of Science from inception to December 2023 to identify studies that examined HR-QoL among patients with scoliosis in China. A list of patient reported outcome measures used in clinical HR-QOL measurement for this population was derived from the selected studies. Stage 2: Reporting followed the PRISMA (Preferred Reporting Items for Systematic Reviews and Meta-Analyses) and AMSTAR (Assessing the methodological quality of systematic reviews) Guidelines. First, we used the COSMIN checklist to assess the methodological quality of studies that reported the measurement properties of the patient reported outcome measures used. Second, the COSMIN tool was used to assess the measurement properties of these instruments, and ratings (excellent, good, fair, or poor) were assigned for each measurement property. Finally, the best evidence was synthesized.

**Results:**

In total, 155 studies were included, and 47 instruments were identified. The top five frequently used HR-QoL instruments for patients with scoliosis in China were the C-SRS-22, ODI, VAS, SF-36, and TC-SRS-22r. The measurement properties assessed included internal consistency (12 instruments), reliability (13 instruments), construct validity (9 instruments), ceiling and floor effects (8 instruments), and responsiveness (3 instruments). The C-ISYQOL had the best methodological quality. No studies assessing measurement properties of instruments for adults with scoliosis were found.

**Conclusions:**

Based on limited research evidence, the C-ISYQOL currently demonstrates the best measurement performance among HR-QoL assessment instruments for Chinese scoliosis patients. However, due to potential publication bias resulting from the restriction of literature searches to published works (excluding grey literature), as well as minor impacts that strict adherence to COSMIN guidelines may still exert on measurement evaluations due to their inherent limitations, the number of studies supporting the favorable measurement properties of the C-ISYQOL remains insufficient. Therefore, it cannot currently be recommended as the preferred instrument for clinical measurement. Although the C-SRS-22 is widely used and has good reliability and validity for HR-QoL assessment in adolescent patients with scoliosis, its measurement properties have not been adequately evaluated in adult populations, and it is not currently supported for use in HR-QoL assessment of adults with scoliosis. Further studies assessing the measurement properties of these instruments are urgently needed.

## Introduction

Scoliosis can be categorized into two main groups based on study populations: adolescent and adult scoliosis. Adolescent idiopathic scoliosis is the most common form of scoliosis (70%–80%) [1]. It has a prevalence of 0.47%–5.2% in the general adolescent population, and affects 2%–4% of adolescents [2]. The prevalence of scoliosis in people aged ≥40 years is 8.85% [3]. Furthermore, the condition is especially prevalent in people aged >65 years, affecting 32%–68% of the population [4]. Scoliosis has a range of reported and comorbidities and can lead to changes in physical appearance, impaired functional mobility, pain, social and psychological burden, and cardiorespiratory function problems, along with significant deterioration in quality of life [5,6]. In clinical practice, the degree of scoliosis correction and recovery of balance are often the objective basis for evaluating treatment efficacy, but patients’ subjective feelings (e.g., quality of life and life satisfaction) are generally neglected. The World Health Organization defines health-related quality of life (HR-QoL) as “an individual’s perception of his or her position in life in the context of the culture and value system in which he or she lives and in relation to his or her goals, expectations, standards and concerns” [7]. HR-QoL among patients with scoliosis has received increased attention from clinicians. Therefore, an effective HR-QoL instrument is an important indicator for assessing patients’ quality of life and the efficacy of clinical interventions for scoliosis, especially for individuals who choose to undergo surgery [8].

The assessment of HR-QoL for patients with scoliosis in China started relatively late and a unified understanding of assessment instruments is lacking, which limits research on quality of life and reduces the credibility of associated interventions for this population. In 1999, Haher et al. developed the Scoliosis Research Society (SRS) instrument [9], which was later improved by Asher et al. (SRS-22) [10]. This improved tool is now widely used in clinical assessment. The SRS-22 was designed to assess HR-QoL in adolescents with idiopathic scoliosis and subsequently became the outcome measure of choice for adult patients. In 2008, Li et al. translated the English version of the SRS-22 into Chinese and performed cross-cultural adaptation to obtain a simplified Chinese version of the SRS-22 (C-SRS-22) [11]. In addition, a large number of measurement instruments, such as the 36-item Short Form Health Survey (SF-36), ODI, and JOA have been used to assess HR-QoL among patients with scoliosis in China. Some of these instruments have been derived from simplified or traditional language versions because of economic and cultural differences in different regions. However, no previous study characterized the HR-QoL of patients with scoliosis in China in terms of the types of outcome measures used, frequency of use, and quality of measurement characteristics.

The Consensus-based Criteria for the Selection of Instruments for Measuring Health Status (COSMIN) is a quality assessment instrument for assessing methodological quality in studies using standardized measurements [12,13]. In 2010, Mokkink et al. conducted an international Delphi study and proposed the COSMIN Inventory, which comprised 10 domains: internal consistency, reliability, measurement error, content validity, structural validity, hypothesis testing, cross-cultural validity, criterion validity, responsiveness, and overall interpretability. The 2012 COSMIN Inventory assessment tool proved to be credible in 46 systematic evaluations [14].

In this study, we conducted a systematic evaluation of the literature on HR-QoL in Chinese patients with scoliosis. We aimed to examine the selection and frequency of use of HR-QOL measurement instruments for Chinese patients with scoliosis in the English-language literature published to date. We also applied the COSMIN scoring tool to systematically assess the measurement properties of the identified instruments.

## Method and materials

### Design

This systematic review used the updated COSMIN methodology for systematic reviews of patient reported outcome measures (PROMs). This systematic evaluation followed the newly revised Preferred Reporting Items for Systematic Evaluation and Meta-Analysis (PRISMA) guidelines and the Assessing Methodological Quality in Systematic Evaluation (AMSTAR) guidelines [15,16,17].The protocol for this review was registered in PROSPERO(CRD42024558825).

### Search strategy

The search strategy used in this study was developed and refined by multiple authors. The MEDLINE, EMBASE, Scopus, PubMed, and Web of Science databases were searched from inception to December 2023. Key search terms and synonyms were searched separately in MESH using four main filters, which were then combined. These filters were: 1) Construct: quality of life OR health-related quality of life OR QoL OR HRQoL; 2) Target population: scoliosis OR adolescent scoliosis OR adolescent idiopathic scoliosis OR adult scoliosis OR adult idiopathic scoliosis OR adult degenerative scoliosis OR degenerative lumbar scoliosis OR spine deformity; 3) Measurement instrument: questionnaire/performance test/measure/instrument/assessment/index OR objective test/measure/assessment OR observational test/measure/assessment/index OR task performance and analysis; and 4) Measurement properties: instrument development OR psychometrics OR clinimetrics OR validity OR reliability OR responsiveness OR interpretability OR meaningful change. Finally, we searched the reference lists of related articles to identify additional potentially relevant literature.

### Eligibility criteria

Retrieved studies initially underwent title and abstract screening to exclude irrelevant studies. The remaining studies were then subject to independent screening by two authors, followed by a full-text review of potentially eligible studies. Any disagreements were resolved through consensus or discussion with an experienced third author. In cases where multiple articles reported the same study results, only the original article was included unless subsequent articles contained new content or results. Studies were included if they were: quantitative studies, including randomized controlled studies and observational studies. Letters, editorials, and conference abstracts were excluded.

### Participants

Individuals diagnosed with scoliosis (Cobb angle >10°; age was not limited).

### Outcome measures

Any studies assessing HR-QoL and relevant PROMs among patients with scoliosis in China.

### Language

Published literature in the English language.

### Data extraction

Data were extracted using a standardized form that included the study sample size, average age of the target population, gender, country, language, and scale parameters. A COSMIN-recommended Excel file is available on the COSMIN website for documenting and determining the quality ratings for each study.

### Missing data

If data were missing or data for patients with scoliosis were reported along with data for other conditions, we contacted the original authors to obtain the necessary information.

### Evaluation of measurement properties of tools used in selected studies

The COSMIN scoring tool was used to assess the quality of the measured attributes reported in each study. Each measured attribute was assigned a separate rating (adequate, inadequate, uncertain). Depending on the number of measurement attributes assessed in a study, some studies underwent one quality assessment, whereas others studies underwent multiple quality assessments.

### Evaluation of the methodological quality of selected studies

The methodological quality of the included studies was evaluated using the COSMIN checklist, which categorizes studies using four quality levels (poor, fair, good, and excellent). The total score for each property is determined by the lowest score. The results were independently assessed by two authors, and any discrepancies were resolved by discussion with a third experienced author.

### Best evidence synthesis: Levels of evidence

In addition to assessing the level of evidence for each of the measured attributes in the instruments used in the selected studies, we integrated the best evidence for three aspects: 1) methodological quality as assessed by the COSMIN tool, 2) assessment of measured attribute results, and 3) the criteria established by Terwee et al. [18]. For all outcome measures, the results for each measured attribute were quantitatively summarized if there was sufficient clinical and methodological homogeneity. If there was a high degree of heterogeneity between studies, a qualitative summary was prepared.

## Results

### Description of included studies and performance-based measures

**Fig 1:**
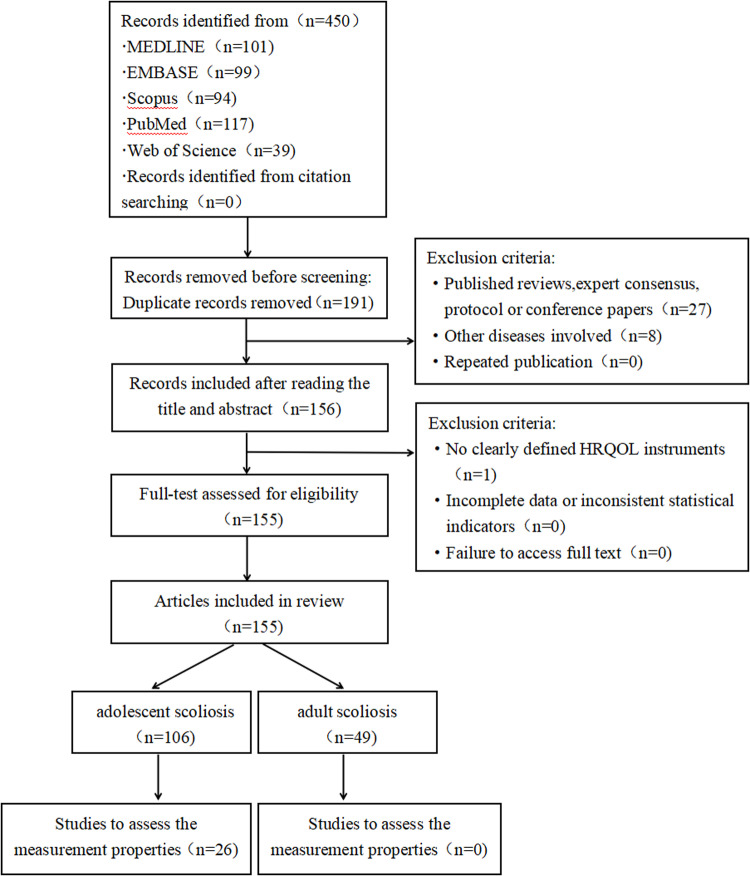
Study flowchart.

In total, 450 relevant papers were retrieved: 101 from MEDLINE, 99 from EMBASE, 94 from Scopus, 117 from PubMed, and 39 from the Web of Science. The research flow chart is shown below (Fig 1).

**Fig 2:**
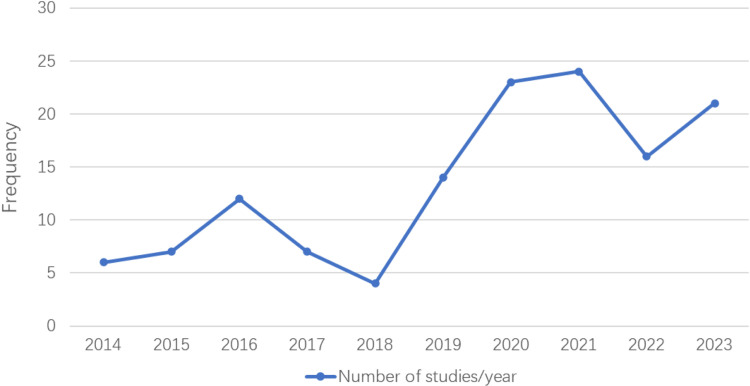
Trend of published English language studies on health-related quality of life among patients with scoliosis in China over the last 10 years.

After screening, 155 published English language studies were included in our analysis. The trend for HR-QoL studies conducted in Chinese patients with scoliosis over the last 10 years is shown in Fig 2. A total of 47 instruments were identified, which were divided into five categories: general instruments (13 instruments), scoliosis-specific instruments (22 instruments), Specific body parts instruments (4 instruments), mental health instruments (6 instruments), and pain instruments (2 instruments). Of these, 9 instruments were used in both adult and adolescent patients with scoliosis, 32 instruments were only used for the adolescent population, and 6 instruments were only used for adults (Table 1).

**Table 1:** Use and frequency of quality of life instruments for people living with scoliosis in China.

Instrument	Frequency	Instrument	Frequency
Generic			
SF-36	27	SF-6D	1
SF-12	2	TC-SF-12	1
PEDSQL4.0:Pediatric Quality of Life Inventory	3	LSIZ:Life Satisfaction IndexZ	2
ICF:International Classification of Functioning	1	CHQ-PF50:Child Health Questionnaire-Parent Form-50	1
C-CHU9D:Child Health Utility 9D	1	GCQ:General Comfort Questionnaire	1
COMI:Core Outcome Measures Index	1	PODCI:pediatric outcomes data collection instrument	1
MBQ:Modifed Baecke Physical Activity Questionnaire	2		
Scoliosis Specific			
C-SRS-22	78	TC-SRS-22	1
C-SRS-22r	16	SRS-20	1
SRS-30	4	C-BRQ	6
TC-EQ-5D-3L	2	TC-EQ-5D-5L	11
TC-EQ-5D-Y-3L	7	TC-EQ-5D-Y-5L	7
C-BSSQ-Brace	1	C-BSSQ-Deformity	1
C-EOSQ-24	2	TC-EOSQ-24	1
C-SAQ	6	TC-SAQ	2
C-ISYQOL	2	TC-ISYQOL	1
BIDQ-S:Body Image Disturbance Questionnaire -Scoliosis	1	QLPSD:quality of life profile for spine deformities	2
TAPS:Trunk Appearance Perception Scale	2	C-EOSQ-SELF	1
Specific body parts			
ODI	52	JOA	11
LSDI:The Lumbar Stiffness Disability Index	3	RMDQ:Roland-Morris Disability Questionnaire	5
Psychosocial and mental health			
BDI-II:Beck Depression Inventory-II	1	DASS:Depression and Stress Scale	1
SES:self-esteem scale	1	STAI:State Trait Anxiety Inventory	1
GAD-7:Generalized Anxiexy Disorde-7	2	PHQ-9:Patient Health Questionnaire-9 items	1
Pain			
VAS	43	NPRS	2

**Fig 3:**
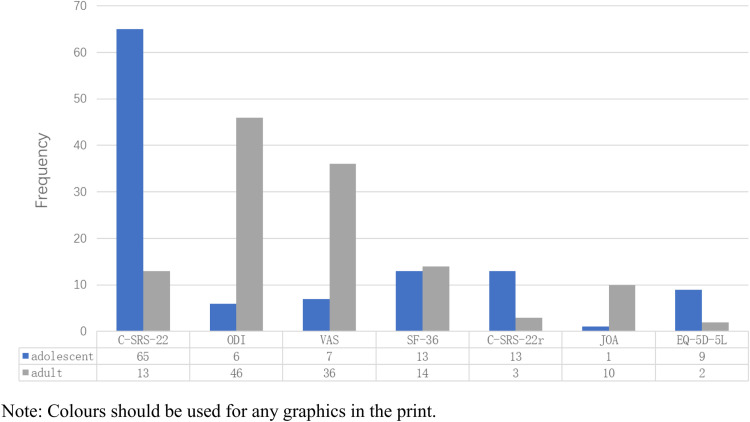
Frequency of use and population distribution of health-related quality of life instruments used in Chinese scoliosis studies.

Three of the 47 identified instruments were used 40 times or more, and 4 instruments were used more than 20 times. The top five most frequently used instruments were: 1) C-SRS-22, 2) ODI, 3) VAS, 4) SF-36, and 5) C-SRS-22r. These five instruments were used 216 times, accounting for 66.6% of the instrument use. The distribution of instruments used more than 10 times in adults and adolescents with scoliosis is shown in Fig 3.

The overall assessment content was divided into seven key domains: physical status, perceived appearance, social functioning, psychosocial and mental health, disease status, treatment satisfaction, and economic status. In the 47 identified instruments, physical status was assessed by physical functioning and mobility (e.g., patients’ tolerance of stairs and strenuous exercise). However, scoring criteria varied, with 2 instruments assessing sexuality and 11 instruments considering sleep or rest. 17 instruments assessed patients’ perceptions of their self-appearance, of which 4 were scoliosis appearance-specific instruments. 25 instruments included assessment of social functioning, such as family relationships, work or study, socialization, and recreational activities. 31 instruments assessed psychosocial and mental health, primarily anxiety, depression, stress, and well-being. Disease status evaluation included assessment of pain, surgery, and degree of scoliosis, with 28 instruments assessing pain (including two pain-specific instruments), in addition to 3 instruments that assessed patients’ postoperative situation, and 2 instruments that assessed scoliosis severity (degree of scoliosis expressed by images of the different degrees of deformity versus the patient’s textual representations of their expectations of the appearance). 7 instruments identified financial burden as an important factor that affected patients’ HR-QoL (see Table 2 for details).

**Table 2:** Assessment content of the generic instruments, scoliosis-specific instruments, and instruments focused on specific body parts.

	Physical condition				appearance	Social function				Mental health		Disease			Treatment satisfaction	Economic
Instrument	General state of health	Body function/motor function	Sex	Sleep/rest	/	Family relationship	Social interaction	Work/study	recreation	Depression/anxiety/stress	happiness	Degree of scoliosis	Pain	operation	/	/
C-SRS-22/TC-SRS-22	–	+	–	–	+	–	+	+	+	+	+	–	+	–	+	+
SRS-20	–	+	–	–	+	–	–	–	–	+	+	–	+	–	+	–
SRS-30	+	+	–	–	+	–	+	+	+	+	+	–	+	+	+	+
C-SRS-22r	–	+	–	–	+	–	+	+	+	+	+	–	+	–	+	+
ODI	–	+	+	+	–	–	+	–	+	–	–	–	+	–	–	–
JOA	–	+	–	+	–	–	–	+	–	–	–	–	+	–	–	–
SF-36	+	+	–	–	–	–	+	+	–	+	+	–	+	–	–	–
C-SF-12/TC-SF-12	+	+	–	–	–	–	+	+	–	+	+	–	–	–	–	–
SF-6D	+	+	–	–	–	–	+	+	–	+	–	–	+	–	–	–
C-EOSQ-24/TC-EOSQ-24	+	+	–	–	–	–	–	–	–	+	–	–	+	–	+	+
C-EOSQ-SELF	+	+	–	+	+	+	+	+	+	+	+	–	+	–	–	–
CHQ-PF50	+	+	–	–	–	+	–	–	–	+	+	–	+	–	–	–
ICF	+	+	–	+	+	+	+	+	+	+	+	–	+	–	+	+
LSIZ	–	–	–	+	–	–	–	+	–	+	+	–	–	–	–	–
TC-EQ-5D(3L/5L)	+	+	–	–	–	–	–	–	–	+	–	–	+	–	–	–
TC-EQ-5D-Y(3L/5L)	+	+	–	–	–	–	–	–	–	+	–	–	+	–	–	–
C-SAQ/TC-ASQ	–	–	–	–	+	–	–	–	–	–	–	+	–	+	–	–
LSDI	–	+	+	–	–	–	–	–	–	–	–	–	–	–	–	–
RMDQ	–	+	–	+	–	–	–	–	–	+	–	–	+	–	–	–
PEDSQL4.0	–	+	–	–	–	–	+	+	+	+	–	–	–	–	–	–
C-BSSQ-Brace	–	–	–	–	+	–	+	–	+	+	–	–	–	–	–	–
C-BSSQ-Deformity	–	–	–	–	–	–	+	–	+	+	+	–	–	–	–	–
C-BIDQ-S	–	+	–	–	+	–	+	+	+	+	+	–	+	–	+	–
COMI	+	+	–	–	–	–	+	+	+	–	–	–	+	–	–	–
C-ISYQOL/TC-ISYQOL	+	+	–	–	+	–	–	–	–	+	+	–	+	–	–	–
C-QLPSD	–	+	–	+	+	–	+	–	+	+	–	–	+	–	–	–
C-BRQ	+	+	–	+	+	+	+	+	+	+	+	–	+	–	+	–
C-CHU9D	–	+	–	+	–	–	–	+	–	+	–	–	+	–	–	–
TAPS	–	–	–	–	+	–	–	–	–	–	–	–	–	–	–	–
GCQ	+	+	–	+	–	+	+	–	–	+	+	–	+	–	–	–
MBQ	+	+	–	+	–	–	–	+	+	–	–	–	–	–	–	–
PODCI	+	+	–	–	+	–	–	–	+	–	+	–	+	–	+	–

## Measurement properties

### Internal consistency

Twenty studies assessed the internal consistency of 12 instruments (see Table 3 for details). Five of these studies reported the internal consistency of the C-SRS-22 [11,19–22], one study gave sufficient evidence (Cronbach’s α = 0.70–0.88), and the remaining four studies did not find statistically significant internal consistency (Cronbach’s α = 0.44–0.89). The internal consistency of the C-ISYQOL and TC-ISYQOL were assessed by one study each, with sufficient evidence (Cronbach’s α = 0.75–0.90) [23,24]. One study used Spearman’s correlation analyses to assess the internal consistency of the C-SAQ [25], and therefore the evidence was unable to be assessed using the COSMIN methodology. One study reported sufficient evidence of internal consistency for the C-BIDQ-S (Cronbach’s α > 0.80) [26]. The internal consistency of the C-EOSQ-SELF [27]was assessed by one study, but the exact values for each subscale were not reported, meaning the adequacy of the evidence could not be assessed. Two studies provided sufficient evidence of internal consistency for the C-QLPSD (Cronbach’s α = 0.75–0.917) [28,29]. The adequacy of internal consistency evidence for C-CHU9D [30] could not be evaluated as precise values were not provided in the study conclusions. The results of the studies evaluating internal consistency for the remaining instruments showed insufficient or conflicting evidence.

**Table 3:** Measurement properties of performance-based measures: Internal consistency.

Instrument	References	Patients	Measurement	Results	Quality	COSMIN
C-SRS-22	Li M, Wang CF,et al. [11]	87	Cronbach’s α	Function/activity subscale(0.81), pain subscale(0.88),Self-image subscale(0.76),Mental health subscale(0.79),Satisfaction subscale (0.65)	–	Fair
	Cheung KM, Senkoylu A, et al. [19]	50	Cronbach’s α	Function/activity、pain and Mental health subscale(0.80–0.89), Self-image and Satisfaction subscale (0.50–0.79)	–	Fair
	Zhao L, Zhang Y, et al. [20]	86	Cronbach’s α	Function/activity subscale(0.70), pain subscale(0.80),Mental health subscale(0.88),Self-image subscale (0.80),Satisfaction subscale(0.81)	+	Fair
	Xie J J, Liu Z D [21]	38	Cronbach’s α	Function/activity、pain、Mental health、Self-image subscale all exceeded 0.70,0.44 for Satisfaction subscale	–	Fair
	Qiu G, Qiu Y, et al. [22]	333	Cronbach’s α	Function/activity subscale(0.57), pain subscale(0.73),Self-image subscale (0.71),Mental health subscale(0.79),Satisfaction subscale (0.50)	–	Excellent
C-ISYQOL	Liu S, Liang J, et al. [23]	138	Cronbach’s α	0.75 for Untreated group,0.88 for Treatment group	+	Excellent
TC-ISYQOL	Cheng AY, Jim PK, et al. [24]	133	Cronbach’s α	Spinal health subscale(0.89),brace subscale(0.79),Cronbach α of 0.89 and 0.90 for participants who answered 20 items and 13 items	+	Fair
C-SAQ	Wei X, Zhu X, et al. [25]	92	Spearman	intradomain correlations ranging from r = 0.526 to r = 0.808 (P, 0.0001)	/	Fair
C-BIDQ-S	Bao H, Yan P, et al. [26]	100	Cronbach’s α	exceeded 0.80 in all subscales	+	Excellent
C-EOSQ-SELF	Yang H, Liu L, et al. [27]	101	Cronbach’s α and McDonald ω	Cronbach α = 0.942,McDonald ω= 0.940 for all subscales	/	Excellent
C-QLPSD	Hou X, Liu S, et al. [28]	172	Cronbach’s α	Cronbach α of all subscales (0.803–0.888),0.917 for whole instrument	+	Fair
	Hu M, Cai Z, et al. [29]	129	Cronbach’s α	Cronbach’s α exceed than 0.75 in all subscales,0.914 for whole instrument	+	Fair
C-CHU9D	Yang P, ChenG, et al. [30]	1912	Cronbach’s α	0.77 for whole instrument	/	Fair
C-BrQ	Liu S, Zhou G, et al. [31]	70	Cronbach’s α	General health perception subscales (0.399),physical function subscales (0.689),Emotional subscales (0.611),Self-esteem and aesthetics subscales(0.791),Vitality subscales (0.646),School activity subscales (0.667),Bodily pain subscales (0.871),Social functioning subscales (0.672)	–	Fair
	Yi H, Chen H, et al. [32]	79	Cronbach’s α	General health perception subscales (0.739),physical function subscales (0.793), Emotional subscales (0.668),Self-esteem and aesthetics subscales (0.812),Vitality subscales (0.677),School activity subscales (0.659),Bodily pain subscales (0.850),Social functioning subscales (0.705)	–	Fair
	Zhang X, Wang D, et al. [39]	208	Cronbach’s α	General health perception、Emotional subscales、Vitality、School activity、Bodily pain and Social functioning subscales(0.70–0.79), physical function、Self-esteem and aesthetics subscales (0.83–0.85)	+	Fair
TC-SAQ	Guo J, Lau AH, et al. [33]	112	Cronbach’s α	General subscale(0.665),shoulder subscale(0.421),Remaining subscale (0.785–0.940)	–	Fair
C-EOSQ-24	Gao R, Sun B, et al. [34]	38	Cronbach’s α	Cronbach’s α exceed than 0.7 in 7 of the 11subscales	–	Excellent
	Li Z, Yue Y, et al. [41]	63	Cronbach’s α	0.950 for 24 subscales,0.927for 11 subscales	+	Good
TC-EOSQ-24	Cheung JPY, Cheung PWH, et al. [40]	100	Cronbach’s α	Quality of life, family burden and satisfaction subscale(0.829–0.919), all subdomains(0.589–0.930)	–	Fair

Rating of each measurement property quality using the COSMIN criteria where “+” is positive, “?” is indeterminate, and “−” is negative.

### Reliability

Twenty-one studies assessed the reliability of 13 instruments (see Table 4 for details). Five studies showed fairly high reliability (intraclass correlation coefficient [ICC] = 0.74–0.96) for the C-SRS-22 [11,19–22]. There was sufficient evidence of retest reliability for the C-ISYQOL and TC-ISYQOL (one study each) [23,24]. Accurate ICC values for each subscale of the C-SAQ [25] were not reported, and therefore reliability was not rated. Evidence for the retest reliability of the C-QLPSD (ICC = 0.722–0.896, 0.784–0.919) and C-BrQ (ICC = 0.807–0.967, 0.809–0.923) was sufficient [28,29,31,32]. There was good evidence of retest reliability for the C-EOSQ-SELF (ICC = 0.818–0.924) [27], TC-SAQ (ICC > 0.798) [33] and C-EOSQ-24 (ICC = 0.681–0.945) [34] (one study each). None of the studies showed good evidence of retest reliability for the TC-EQ-5D-5L or TC-EQ-5D-Y-5L/3L [35–37]. ICC values for COMI [38] subscales were unreported, precluding reliability assessment.

**Table 4:** Measurement properties of performance-based measures: Reliability.

Instrument	References	Patients	Interval	Design	Measurement	Results	Quality	COSMIN
C-SRS-22	Li M, Wang CF,et al. [11]	63	8-26 days	Test-retest analysis	ICCs	Function/activity subscale (0.74),pain subscale(0.78), Mental health subscale (0.81),Self-image subscale (0.86),Satisfaction subscale (0.84)	+	Good
	Cheung KM, Senkoylu A,et al. [19]	86	2-15 days	Test-retest analysis	ICCs	Function/activity subscale (0.83),pain subscale(0.76), Mental health subscale (0.84),Self-image subscale (0.79),Satisfaction subscale (0.82)	+	Good
	Zhao L, Zhang Y,et al. [20]	30	21-28 days	Test-retest analysis	ICCs	Function/activity subscale (0.85),pain subscale(0.96), Mental health subscale (0.95),Self-image subscale (0.96),Satisfaction subscale (0.91)	+	Fair
	Xie J J, Liu Z D [21]	38	8-18 days	Test-retest analysis	ICCs	Function/activity subscale (0.74),pain subscale(0.78), Mental health subscale (0.81),Self-image subscale (0.86),Satisfaction subscale (0.84)	+	Fair
	Qiu G, Qiu Y, et al. [22]	84	8-21 days	Test-retest analysis	ICCs	Function/activity subscale (0.78),pain subscale(0.70), Mental health subscale (0.82),Self-image subscale (0.85),Satisfaction subscale (0.75)	+	Good
C-ISYQOL	Liu S, Liang J, et al. [23]	70	14 days	Test-retest analysis	ICCs	Untreated group(0.72), Brace treatment group (0.80)	+	Good
TC-ISYQOL	Cheng AY, Jim PK, et al. [24]	133	14 days	Test-retest analysis	ICCs	All subscales(0.95–0.96)	+	Fair
C-SAQ	Wei X, Zhu X, et al. [25]	92	4-7days	Test-retest analysis	ICCs	0.933for whole instrument	/	Fair
C-EOSQ-SELF	Yang H, Liu L, et al. [27]	101	14 days	Test-retest analysis	ICCs	All subscales(0.818–0.924)	+	Fair
C-QLPSD	Hou X, Liu S, et al. [28]	69	14 days	Test-retest analysis	ICCs	All subscales(0.722–0.896)	+	Good
	Hu M, Cai Z, et al. [29]	129	7-14 days	Test-retest analysis	ICCs	All subscales(0.784–0.919)	+	Good
C-BrQ	Liu S, Zhou G, et al. [31]	70	14 days	Test-retest analysis	ICCs	All subscales(0.807–0.967)	+	Good
	Yi H, Chen H, et al. [32]	72	7-12 days	Test-retest analysis	ICCs	All subscales(0.809–0.923)	+	Good
TC-SAQ	Guo J, Lau AH, et al. [33]	112	14 days	Test-retest analysis	ICCs	ICCs exceed than 0.798 in all subscales	+	Excellent
C-EOSQ-24	Gao R, Sun B, et al. [34]	38	14 days	Test-retest analysis	ICCs	0.681 for fatigue and energy levels subscale, ICCs exceed than 0.790 in other subscales	+	Fair
TC- EQ-5D-5L	Cheung PWH, Wong CKH, et al. [35]	106	15-36 days	Test-retest analysis	ICCs	Weighted Kappa Less than 0.70 in all subscales	_	Fair
TC- EQ-5D-Y-5L	Lin J, Wong CKH, et al. [36]	129	118 days	Test-retest analysis	ICCs, GAC, PA	ICCs Less than 0.40 in EQ-VAS subscale	/	Good
	Wong CKH, Cheung PWH, et al. [37]	110	90 days	Test- retest analysis	Gwet	/	/	Poor
TC- EQ-5D-Y-3L	Lin J, Wong CKH, et al. [36]	129	118 days	Test-retest analysis	ICCs, GAC, PA	ICCs Less than 0.40 in EQ-VAS subscale	/	Good
	Wong CKH, Cheung PWH, et al. [37]	110	90 days	Test-retest analysis	Gwet	/	/	Poor
COMI	Qiao J, Zhu F, et al. [38]	120	30 days	Test-retest analysis	ICCs	0.91for whole instrument,Pain subscale of back and legs(0.81–0.86)	/	Good

ICC, intraclass correlation coefficient. Rating of each measurement property quality using the COSMIN criteria where “+” is positive, “?” is indeterminate, and “−” is negative.

### Validity

Thirteen studies assessed the construct validity of nine instruments (see Table 5 for details). The structural validity of the C-SRS-22 was poor, with only one of the five studies rating the measurement properties as good [11,19–22]. The remaining eight instruments had good construct validity, but each was only supported by one study [23,24,29,33,35,38–40]. There were no studies assessing criterion validity,cross-cultural validity, or hypothesis testing.

**Table 5:** Measurement properties of performance-based measures: structural validity.

Instrument	References	Patients	Design	Result	Correlation	Quality	COSMIN
C-SRS-22	Li M, Wang CF et al. [11]	63	Pearson’s correlation	Function/activity subscale (0.38–0.76),pain subscale (0.30–0.81),Mental health subscale(0.32–0.85),Self-image subscale(0.17–0.51), Satisfaction subscale(0.16-0.32)	SF-36	_	Fair
	Cheung KM, Senkoylu A,et al. [19]	50	Pearson’s correlation	Function/activity subscale (0.59–0.77),pain subscale (0.54–0.72),Mental health subscale(0.57–0.67),Self-image subscale(0.50–0.62), Satisfaction subscale(0.25-0.49)	SF-36	_	Fair
	Zhao L, Zhang Y, et al. [20]	86	CFA	Function/activity subscale (0.416–0.819),pain subscale (0.707–0.794),Mental health subscale(0.616–0.813),Self-image subscale(0.762–0.892),Satisfaction subscale (0.917)	/	+	Fair
	Xie J J, Liu Z D [21]	38	Pearson’s correlation	Function/activity subscale (0.328–0.576),pain subscale (0.166–0.665),Mental health subscale(0.011–0.553),Self-image subscale(0.018–0.553),Satisfaction subscale(0.164−0.367)	SF-36	_	Fair
	Qiu G, Qiu Y, et al. [22]	333	Pearson’s correlation	Function/activity subscale (0.42–0.70),pain subscale (0.30–0.52),Mental health subscale(0.36–0.62),Self-image subscale(0.22–0.63), Satisfaction subscale(0.16-0.32)	SF-36	_	Fair
C-ISYQOL	Liu S, Liang J, et al. [23]	138	Pearson’s correlation	r = 0.59–0.72	C-SRS-22	+	Fair
TC-ISYQOL	Cheng AY, Jim PK, et al. [24]	133	Rasch	total variance = 45.9%, eigenvalue = 17	/	+	Good
C-QLPSD	Hu M, Cai Z, et al. [29]	129	Pearson’s correlation	r(C-SRS-22)=−0.924, r(SF-36)=−0.871	SF-36 C-SRS-22	+	Fair
TC-SAQ	Guo J, Lau AH, et al. [33]	101	Pearson’s correlation	r = 0.820–0.954	E-SAQ	+	Fair
TC-EQ-5D-5L	Cheung PWH, Wong CKH, et al. [35]	227	Spearman’s correlation	Function/activity subscale (0.72),pain subscale(0.59), Mental health subscale (0.57),Self- image subscale (0.51),Satisfaction subscale (−0.14)	C-SRS-22r	+	Excellent
COMI	Qiao J, Zhu F, et al. [38]	120	Spearman’s correlation	r(VAS)=0.89, r(SF-36)=−0.59–0.84, r(ODI)=0.45–0.72, r(RMQ)=0.31–0.62	ODI、RMQ VAS、SF-36	+	Fair
C-BrQ	Zhang X, Wang D, et al. [39]	208	Pearson’s correlation	r = 0.51–0.83	C-SRS-22	+	Fair
TC-EOSQ-24	Cheung JPY, Cheung PWH, et al. [40]	100	Pearson’s correlation	Quality of life subscale (0.312–0.716),Family burden subscale (0.349−0.725),Satisfaction subscale(0.306–0.702)	C-CHQ-PF D-50	+	Fair

CFA, confirmatory factor analysis. Rating of each measurement property quality using the COSMIN criteria where “+” is positive, “?” is indeterminate, and “−” is negative.

### Floor and ceiling effects

A total of 14 studies assessed the floor and ceiling effects of nine instruments (see Table 6 for details). All C-SRS-22 subscales had varying degrees of ceiling effects [20,22], with these effects ranging from 2.3% to 67.5%. Similar high ceiling effects were observed for the C-BrQ [31,32,39], TC-EQ-5D-Y-3L/5L [36,37,42], TC-EOSQ-24 [40] and C-EOSQ-24 [41]. Floor effects were observed in four of the five C-QLPSD subscales (20.0%–45.7%) [28–29].

**Table 6:** Measurement properties of performance-based measures: responsiveness, floor, and ceiling effects.

Responsiveness							Floor and ceiling effects		
Instrument	References	Patient	Measurement	Result	Quality	COSMIN	Type	Result	Quality
C-SRS-22	Zhao L, Zhang Y, et al. [20]	86	/	/	/	/	floor/ ceiling	2.3%−15.1%/2.3%−7.0%	+
	Qiu G, Qiu Y, et al. [22]	333	/	/	/	/	floor/ ceiling	14.0%−67.5%/1.2%−5.9%	_
C-EOSQ-SELF	Yang H, Liu L, et al. [27]	101	/	/	/	/	floor/ ceiling	0%−52.48%/0%−7.92%	_
C-QLPSD	Hou X, Liu S, et al. [28]	172	/	/	/	/	floor/ ceiling	0%−28.6%/20%−45.7%	_
	Hu M, Cai Z, et al. [29]	129	/	/	/	/	floor/ ceiling	0%−4.65%/0%−0.78%	+
C-BrQ	Liu S, Zhou G, et al. [31]	70	/	/	/	/	floor/ ceiling	0%−45.7%/0%−10%	_
	Yi H, Chen H, et al. [32]	79	/	/	/	/	floor/ ceiling	2.5%−39.2%/0%−12.7%	_
	Zhang X, Wang D, et al. [39]	217	/	/	/	/	floor/ ceiling	0.76%−40.76%/0%	_
TC-EQ-5D-5L	Cheung PWH, Wong CKH, et al. [35]	176	SES、SRM	health worsened subgroup: SES(−0.71),SRM(−0.40);unchanged group:SES (−0.06),SRM(−0.05); health improved subgroup:SES(0.25), SRM(0.23)	+	Fair	/	/	/
TC-EQ-5D-Y-3L/5L	Lin J, Wong CKH, et al. [36]	130	/	/	/	/	ceiling	Y-5L:62.8%−97.7%; Y-3L:62.0%-96.9%	_
	Wong CKH, Cheung PWH, et al. [37]	129	ROC Curve- analysis	AUC = 0.70,95% CI (0.43–0.97)	+	Fair	floor/ ceiling	floor:0%, Ceiling of Y-5L: 58.6%, ceiling of Y-3L:61.2%	_
	Pei W, Yue S, et al. [42]	262	/	/	/	/	ceiling	Ceiling of Y-5L:40.3%, ceiling of Y-3L:44.1%	_
COMI	Qiao J, Zhu F, et al. [38]	120	/	/	/	/	floor/ ceiling	0%−24.2%/5.8%−28.3%	+
TC-EOSQ-24	Cheung JPY, Cheung PWH, et al. [40]	100	/	/	/	/	floor/ ceiling	0%−71.0%/0%−26.0%	_
C-EOSQ-24	Li Z, Yue Y, et al. [41]	63	/	/	/	/	floor/ ceiling	0%−88.9%/0%−3.2%	_

Rating of each measurement property’s quality using the COSMIN criteria, where “+” is positive, “?” is indeterminate, and “−” is negative.

### Responsiveness

Responsiveness was assessed in two studies using SES, SRM (TC-EQ-5D-5L), and receiver operating curve analysis (TC-EQ-5D-Y-5L/3L) (see Table 6 for details). The TC-EQ-5D-5L was responsive to detecting negative changes in the deteriorating health subgroup and mildly responsive in the improving health subgroup, despite a mean score of 0.92 at baseline and a non-significant mean change in the unchanged group [35]. The TC-EQ-5D-Y-3L/5L responded to both “worsening” and “improving” health changes. and to “no change” in health status or treatment modalities. The mean change in the TC-EQ-5D-Y-3L/5L from baseline to a 3-month follow-up was not significant, with an area under the curve of 0.70 and a 95% confidence interval of 0.43–0.97 [37].

## Methodological evaluation of measurement properties

Based on the COSMIN criteria, one study focused on the C-ISYQOL showed the best results [23], with the methodological quality of the measurement properties ranging from excellent to fair. One study focused on the TC-ISYQOL and two studies on the C-QLPSD showed a good or fair methodological quality of measurement properties [24,28,29]. The methodological quality of the measurement properties of the C-SRS-22 ranged from excellent to fair [11,19–22].

## Best evidence synthesis

The overall level of evidence for the 18 instruments is summarized in Table 7. In terms of internal consistency, the quality of evidence was strong for the C-ISYQOL and C-BIDQ-S. In terms of reliability, the TC-SAQ had a strong level, whereas the C-SRS-22, C-ISYQOL, C-BrQ, and C-QLPSD had a moderate level. The TC-EQ-5D-5L had good performance both in terms of construct validity and responsiveness.

**Table 7:** Summary of measurement properties.

Instrument	Internal consistency	Reliability	construct validity	Responsiveness
C-SRS-22	+	++	+	/
C-ISYQOL	+++	++	+	/
TC-ISYQOL	+	+	+	/
C-SAQ	/	/	/	/
TC-SAQ	_	+++	+	/
C-BSSQ-Brace	/	/	/	/
C-BSSQ-Deformity	/	/	/	/
C-BIDQ-S	+++	/	/	/
TC-EOSQ-24	_	/	+	/
C-EOSQ-24	+/-	+	/	/
C-EOSQ-SELF	/	+	/	/
C-BrQ	+/-	++	+	/
C-QLPSD	++	++	+	/
C-CHU9D	/	/	/	/
TC-EQ-5D-5L	/	_	+++	+
TC-EQ-5D-Y-5L	/	/	/	+
TC-EQ-5D-Y-3L	/	/	/	+
COMI	/	/	+	/
C-EOSQ-SELF	/	+	/	/

Note: +++ Consistent positive evaluation results in multiple studies of “good” methodological quality, or in one study of “excellent” methodological quality; ++ Consistently positive ratings across multiple studies of “fair” methodological quality or one study of “good” methodological quality; + positive results were obtained in a study of “fair” methodological quality; + /- conflicting results;? only studies with “poor” methodological quality; − a study with “fair” methodological quality was negative.

## Discussion

The impact of scoliosis on patients’ HR-QoL is significant. Currently, there is no high-level evidence to support any treatment that can prevent adolescent scoliosis from progressing into adulthood or improve patients’ long-term HR-QoL. Scoliosis in adolescents is likely to progress with age, and treatment aims to reverse, prevent, or limit further progression to prevent or reduce symptoms. Braces are the most common conservative treatment for patients with mild-to-moderate malformations, and surgical intervention is required when the Cobb angle exceeds 40° [43]. In addition to affecting normal physiological activities and appearance, there is a significant correlation between adolescent scoliosis and mental health problems such as depression and anxiety [44].

Adult scoliosis differs from adolescent scoliosis in that the main reason for seeking medical treatment is not appearance deformity, but rather the pain and dysfunction caused by the condition. Treatment for these patients focuses on addressing pain and dysfunction to improve their quality of life. Non-surgical treatments (e.g., physical therapy, hot compresses, stretching, and aerobic exercise) are initially considered first-line treatment, particularly for those with mild baseline disease (as scored using the SRS-22) [45]. HR-QoL is an important indicator for assessing treatment outcomes among patients that opt for surgery. In the last decade there has been a dramatic shift in the philosophy of both doctors and patients regarding the treatment of adult scoliosis, with an increase in the number of individuals opting for surgical treatment; however, surgical complications occur at a rate of up to 40% and affect nearly 70% of patients [46,47]. Such complications result in significant deterioration in health-related quality of survival. Recent studies have shown that 27%–38% of adult patients with scoliosis have comorbid mental health disorders (e.g., concomitant depression, sleep, anxiety, and stress disorders), and are more likely to experience surgical complications and revision for at least 2 years after surgery [48,49].

The current status of HR-QoL instruments used to evaluate patients with scoliosis in China is not clearly defined because many different instruments are available for this purpose. In this study, we identified commonly used HR-QoL instruments and the frequency of use of these tools in Chinese patients with scoliosis based on published English literature. We also evaluated the measurement properties of these instruments using the COSMIN approach. In total, 47 instruments were identified, among which the C-SRS-22, ODI, VAS, SF-36, and C-SRS-22r were most frequently used. However, most instruments had limited frequency and application range or insufficient quality of evidence of good measurement properties. For example, although the C-ISYQOL had good measurement properties based on one high-quality study, this may not be sufficient to recommend it as a preferred instrument for clinical measurement because of a lack of supporting evidence. Furthermore, as research on the C-ISYQOL increases, its measurement properties may be downgraded in further studies. The widely used SF-36 lacks specificity for patients with scoliosis and takes too long to evaluate because of repeated questions. Currently, the SRS strongly recommends using the SRS-22 and SRS-22r globally for evaluating HR-QoL among patients with scoliosis. The measurement properties of the SRS-22 have been evaluated in Spain, Italy, Turkey, and other countries and regions, and the results showed that it has good reliability and validity for evaluating HR-QoL in patients with scoliosis. The results of this study also showed that the C-SRS-22 had good properties in the measurement of HR-QoL in Chinese adolescent patients with scoliosis.

It is worth noting that the measurement properties of the abovementioned instruments were all examined in adolescent populations, and no studies were found that assessed measurement instruments in adult patients with scoliosis. This was consistent with international findings. For example, Archer [50] conducted a systematic review of the literature published in English that assessed HR-QoL in adult scoliosis, but did not include any studies published in Chinese and conducted in China showed that there was insufficient evidence for the measurement properties of any HR-QoL outcome measures in adult scoliosis. The HR-QoL outcome domains in adult patients with scoliosis using the C-SRS-22 may differ compared with those in adolescent scoliosis. There was also a lack of assessment of the content validity of C-SRS-22 in adult scoliosis, which is the most important measurement property for determining the suitability of a measurement instrument for clinical application. Further studies are urgently needed to assess the measurement properties of HR-QoL instruments in adult scoliosis populations.

The findings of this systematic review indicate that the current clinical assessment instruments for HR-QoL in Chinese scoliosis patients present a landscape dominated by generic instruments, with delayed development of disease-specific measures. The C-SRS-22, ODI, VAS, SF-36, and JOA scores constitute the primary evaluation system in clinical research. This situation reflects two underlying issues: First, the localization process of scoliosis-specific assessment instruments in Chinese lags significantly. Apart from the SRS-22, internationally recognized instruments such as the Quality of Life Profile for Spine Deformities (QLPSD) and Brace Questionnaire have not yet established validated Chinese versions. Second, there is an overemphasis on pain and functional disability dimensions, with predominant use of ODI and VAS combinations, potentially underestimating patients’ specific needs in multidimensional health domains such as social participation and psychological well-being. Additionally, the validation populations for these instruments’ measurement properties demonstrate significant age bias, which starkly contrasts with China’s epidemiological trend of continuously rising prevalence of adult degenerative scoliosis (ADS).

Based on the differentiated evaluation needs of clinical treatment approaches, conservative treatment emphasizes process monitoring and requires high-sensitivity measurement instruments, while surgical treatment prioritizes outcome evaluation and demands multidimensional and specific scales. For adult spinal deformity management, it is essential to integrate degeneration-related indicators. Current strategies for selecting assessment instruments, tailored to treatment modalities, can be established as follows:

a Conservative Treatment (Bracing/Rehabilitation) Recommended Combination: It is recommended to adopt an evaluation model combining the C-ISYQOL with dynamic VAS scores. This study found that, under the COSMIN criteria, the C-ISYQOL demonstrates superior overall measurement properties—including internal consistency, reliability, and structural validity—compared to the C-SRS-22. Its unique dimensions, such as “Treatment Compliance Distress” (Item 15) and “Dynamic Body Image Perception” (Items 7–9), effectively capture critical turning points during brace therapy. Additionally, dynamic VAS scoring is recommended every 3 months as a sensitivity supplement.
b Surgical Treatment (Fusion/Non-fusion) Recommended Combination: A three-tiered assessment system combining the C-SRS-22, ODI scores, and SF-36 scores is proposed. The SRS-22 questionnaire is endorsed by international scoliosis research consortia (e.g., SRS, ESSG) as the standard postoperative follow-up instrument, with no other scoliosis-specific scale currently addressing the postoperative evaluation needs of both adolescent and adult populations. The ODI should focus on pain-related dysfunction during the critical recovery period (6 weeks to 3 months postoperatively) [51], while the SF-36 serves as a long-term follow-up supplement (≥1 year), particularly for evaluating the impact of adjacent segment degeneration on overall health.
c Adult Spinal Deformity Management Strategy: There is an urgent need to develop composite scales integrating degeneration-specific metrics. Existing instruments exhibit significant “dimensional gaps” in assessing HRQoL in adult scoliosis patients. For instance, the C-SRS-22 lacks specificity for degenerative spinal symptoms (e.g., radicular pain, neurological deficits, spinal stenosis) and prioritizes daily activities (e.g., dressing, lifting) over critical adult-specific concerns such as gait stability, fall risk, prolonged standing/walking capacity, and comorbidities (e.g., osteoporosis, arthritis). While the ODI overemphasizes low back pain, the Core Outcome Measures Index (COMI)—validated for lumbar degenerative conditions and adopted by EuroSpine’s Spine Tango registry—is recommended as a transitional short-term solution [52].

In addition, further research should explicitly address the mental health of adolescents and adults with scoliosis, including epidemiological studies, systematic evaluations based on big data, and use of specific measurement instruments. Psychosocial interviews and randomized clinical trials should be conducted to assess the effectiveness of psychoeducational and psychotherapeutic interventions for patients suffering psychological distress because of scoliosis. Psychological interventions should form an integral part of the overall treatment program for patients with scoliosis to meet the growing need for psychosocial support for these patients and reduce the social burden of developing mental health problems.

The strength of this systematic evaluation was that it was the first study to assess the methodological quality of and psychometric evidence for HR-QoL measurement instruments in Chinese patients with scoliosis based on the COSMIN criteria, which provides useful evidence for selecting appropriate HR-QoL clinical measurement instruments for Chinese patients with scoliosis. However, the results of this systematic evaluation were limited by the fact that only published literature was searched. Data from unpublished studies were not included in the analyses, although the number of excluded studies was much smaller than the included studies. However, a small amount of missing data could lead to potential publication bias and undermine the validity of the findings. In addition, methodological flaws in the COSMIN methodology may have had a slight effect on the results.

## Conclusion

The C-SRS-22, ODI, VAS, SF-36, and C-SRS-22r are the most commonly used HR-QoL measurement instruments for Chinese patients with scoliosis in the English language literature published to date. Of these, only the C-SRS-22 has been fully evaluated in terms of its measurement properties. Although the C-SRS-22 is a scoliosis-specific HR-QoL instrument, its measurement properties have not been validated in an adult scoliosis population. Among the studies included for COSMIN methodological assessment, the measurement properties of the C-ISYQOL showed the best results, but its generalized use cannot currently be fully supported because only one high-quality study was available. From the perspective of methodological quality, it is difficult to identify a suitable instrument, and further multicenter studies are needed to develop a HR-QoL instrument suitable for Chinese patients with scoliosis based on the Chinese national and cultural context.

**Provenance and peer review:** Not commissioned, externally peer-reviewed.

## Supporting information

S1 File PRISMA checklist. (PDF)
